# Estimation of lung cancer deaths attributable to indoor radon exposure in upper northern Thailand

**DOI:** 10.1038/s41598-022-09122-y

**Published:** 2022-03-25

**Authors:** Kawinwut Somsunun, Tippawan Prapamontol, Chaicharn Pothirat, Chalerm Liwsrisakun, Donsuk Pongnikorn, Duriya Fongmoon, Somporn Chantara, Rawiwan Wongpoomchai, Warangkana Naksen, Narongchai Autsavapromporn, Shinji Tokonami

**Affiliations:** 1 Environment and Health Research Unit, Research Institute for Health Sciences (RIHES), Chiang Mai University, Chiang Mai, 50200 Thailand; 2 PhD Degree Program in Environmental Science, Environmental Science Research Center, Faculty of Science, Chiang Mai University, Chiang Mai, 50200 Thailand; 3 Department of Internal Medicine, Faculty of Medicine, Chiang Mai University, Chiang Mai, 50200 Thailand; 4 Lampang Cancer Hospital, Department of Medical Services, Ministry of Public Health, Lampang, Thailand; 5 Environmental Science Research Center, Faculty of Science, Chiang Mai University, Chiang Mai, 50200 Thailand; 6 Department of Biochemistry, Faculty of Medicine, Chiang Mai University, Chiang Mai, 50200 Thailand; 7 Faculty of Public Health, Chiang Mai University, Chiang Mai, 50200 Thailand; 8 Department of Radiology, Faculty of Medicine, Chiang Mai University, Chiang Mai, 50200 Thailand; 9 Institute of Radiation Emergency Medicine, Hirosaki University, Hirosaki, Aomori 036-8564 Japan

**Keywords:** Risk factors, Lung cancer, Natural hazards

## Abstract

Radon exposure is the second leading cause of lung cancer, after smoking. In upper northern Thailand (UNT), lung cancer incidence was frequently reported by Thailand National Cancer Institute. Besides smoking, radon exposure may also influence the high lung cancer incidence in this region. Indoor radon concentrations were measured in 192 houses in eight provinces of UNT. Indoor radon concentrations ranged from 11 to 405 Bq m^−3^ and estimated annual effective dose ranged from 0.44 to 12.18 mSv y^−1^. There were significant differences in indoor radon concentrations between the houses of lung cancer cases and healthy controls (p = 0.033). We estimated that 26% of lung cancer deaths in males and 28% in females were attributable to indoor radon exposure in this region. Other factors influencing indoor radon levels included house characteristics and ventilation. The open window-to-wall ratio was negatively associated with indoor radon levels (B = −0.69, 95% CI −1.37, −0.02) while the bedroom location in the house and building material showed no association. Indoor radon hence induced the fractal proportion of lung cancer deaths in UNT.

## Introduction

Lung cancer is the leading cause of cancer deaths worldwide. In 2020, there were an estimated 1.8 million lung cancer deaths, accounting for 18% of all cancer deaths globally^1^. In Thailand, lung cancer was a main cause of death with 23,713 cases in 2020, contributing to 12.4% of all cancer deaths^2^. The problem is especially severe in northern Thailand where lung cancer incidence and mortality were twice as high as other areas of the country^3^. Recently, lung cancer incidence in this region has declined, likely as a result of decreased tobacco smoking, the major risk for development of lung cancer^4^. However, lung cancer continues to have significantly higher incidence in northern Thai men and women compared to all other regions, and it is still one of the most causes of cancer death in upper northern Thailand (UNT)^5^.

There are many known risk factors causing lung cancer, particularly, tobacco smoking. In 2004, the International Agency for Research on Cancer (IARC), reported that more than 80% of lung cancer patients were related to tobacco smoking, both voluntary and involuntary^6^. However, more than 25% of lung cancer patients were non-smokers, particularly for women^7^. Lung cancer among non-smokers remains among the top-ten causes of cancer-related death in the world^8^. In northern Thailand, the prevalence of daily tobacco smoking has continually decreased to 18.4% in 2009^5,9^. Concurrently, the types of lung cancer found in UNT have also shifted, with a decline incidence in squamous and small cell lung carcinomas, which are the types most linked to smoking, and increases in adenocarcinomas, which are more weakly linked to smoking but more strongly linked to environmental factors^4,10,11^. Therefore, other environmental factors might play a crucial role in lung cancer development, such as radon gas, air pollution, household smoke, asbestos and occupational risk factors^12–14^.

After smoking, radon is the second most important cause of lung cancer, excluding the genetic and other natural related biological factors. Radon and its progenies are the most important contributors to human exposure to high natural radiation^15–18^ and approximately 10–20% of lung cancer worldwide was a result of radon exposure^19^. Radon (^222^Rn) is a radioactive gas resulting from radium decay (^226^Ra), itself a decay product of uranium (^238^U), which is naturally found on the earth's crust. Radon gas is inert, odorless, tasteless, invisible and can readily emanate and be concentrated in enclosed areas where it is trapped ^17,19^. Most inhaled radon is rapidly exhaled, but inhaled progenies as solid particles are able to readily deposit on the walls of the bronchial epithelium, where it delivers most of the radiation dose. As these progenies emit alpha particles over the short term, these particles can interact with biological molecules in the lung, leading to DNA damage, mutations and ultimately development of cancer^17,19–21^. In 1988, radon has been classified as a known human carcinogen (Group1) by the IARC^22^. In the last several decades, many studies have found the association between lung cancer and long-term exposures to residential radon^23–25^. The induction period of lung cancer attributable to radon exposure in humans is between 5 and 25 years^26^. The high dose and long-term exposure to radon in UNT was a crucial factor that enhanced lung cancer development^14^. Radon is a linear non-threshold carcinogen that can induce the risk of lung cancer without minimal value of concentration^17^. Additionally, the general population study suggests that chronic low dose exposure to radon can cause lung cancer development, for every 100 Bq m^−3^ increase in indoor radon concentration, the risk of lung cancer is estimated to increase by 8–33%^23,24,26,27^. The WHO recommended average annual reference level of indoor radon is currently 100 Bq m^−3^ and it also varies by countries^19^. To elucidate the potential contribution of radon exposure on the high incidence of lung cancer in UNT, the case–control study was conducted to evaluate the relationship between radon exposure and lung cancer incidence in UNT where the research data are scarce.

## Results and discussion

The demographic characteristics of participants are comparable and shown in Table S1 (Supplementary Table S1). The indoor radon concentration of 192 participant bedrooms in the eight provinces of UNT is presented in Table 1. This ranged from 11 to 405 Bq m^−3^, with an arithmetic mean of 105 ± 74 Bq m^−3^ and geometric mean of 80 Bq m^−3^, which is higher than the global average of 39 Bq m^−3^^19^ and the domestic mean of 16 Bq m^−3^ in Thailand^28^. The arithmetic mean was slightly higher than the WHO reference level and lower than the EPA action level of 148 Bq m^−3^. The mean indoor radon concentration showed significant differences (p < 0.001) between the provinces of UNT. The highest indoor radon concentration was found in Phrae province, with a arithmetic mean ± SD (range) of 168 ± 69 (54–286) Bq m^−3^, follow by Phayao, Chiang Rai, Chiang Mai, Nan, Mae Hong Son, Lampang and Lamphun provinces with the mean ± SD (range) of 167 ± 52 (64–219), 139 ± 77 (31–242), 110 ± 87 (16–405), 90 ± 55 (25–207), 84 ± 55 (35–241), 78 ± 54 (32–216) and 75 ± 60 (11–193) Bq m^−3^, respectively.

**Table 1:** Arithmetic and geometric means of indoor radon concentrations in eight provinces of upper northern Thailand (UNT).

Provinces	Houses(n)	Rn concentration (Bq m^−3^)			
		Mean (SD)	Geomean	Min	Max
UNT	192	105 (74)	80	11	405
Phrae	16	168 (69)	152	54	286
Phayao	7	167 (52)	157	64	219
Chiang Rai	25	139 (77)	112	31	242
Chiang Mai	46	110 (87)	84	16	405
Nan	10	90 (55)	75	25	207
Mae Hong Son	17	84 (55)	71	35	241
Lampang	32	78 (54)	65	32	216
Lamphun	39	75 (60)	53	11	193

Of 192 surveyed houses, 41% and 30% had radon concentration higher than the WHO and EPA recommended levels (Table S1) which may be associated with the higher incidence and mortality of lung cancer in UNT compared to other regions of Thailand.

**Figure 1:**
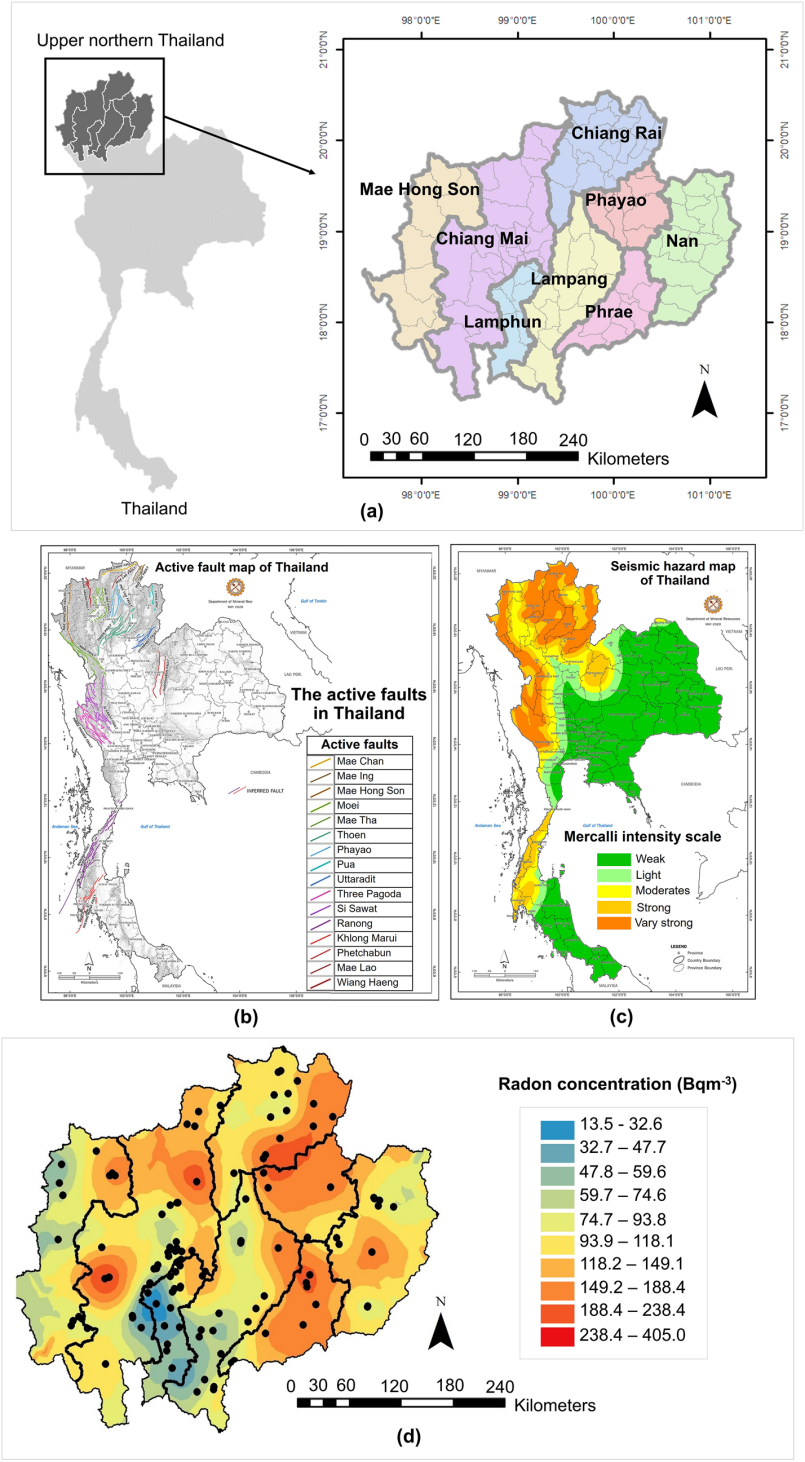
Study area, sample locations and distribution of indoor radon concentrations. (**a**) Study sites of eight provinces in upper northern Thailand, (**b**) Active fault zones in Thailand, (**c**) Seismic hazard map of Thailand, (**d**) Distribution of indoor radon concentration, with sampling points indicated in black dots using ArcMap software, Geostatistical wizard, Kriging method. Active fault zones in Thailand map and Seismic hazard map of Thailand obtained from the Department of Mineral Resources, Thailand (http://webeng.dmr.go.th/Show_Detail.aspx?DetailId=97).

The distribution of indoor radon concentrations and measurement locations in UNT is shown in Fig. 1. To estimate the indoor radon value for all eight provinces, the geostatistical Kriging interpolation was used to create a radon distribution map. As the UNT region is located in different radon potential basin areas of granite, there is abundant uranium and its decay products around this area^29,30^. Reportedly, granitic gneiss has high frequency ratios for radon levels^31,32^.

UNT is also located in the area of nine active fault zones (Fig. 1b,c). Faults and fractures can preferentially release the radon gas to the surface^33,34^ and can enhance radon concentration by fault and seismic activity^35–38^. Therefore, the presence of active fault zones may contribute to high radon concentrations in the UNT region relative to the rest of the country.

When comparing the mean indoor radon concentration in this study with others conducted in UNT (Table 2), we found more than 80% of houses had indoor radon concentration higher than the global average (39 Bq m^−3^). Moreover, the concentrations found in this study were also higher than the mean value of 16 Bq m^−3^ for Thailand^28^.

**Table 2:** Arithmetic means indoor radon concentrations previously reported in upper northern Thailand (UNT).

Area	Detector		Study design	Indoor Radon (Bq m^−3^)	References
				Arithmetic mean (SD), case/control	
	n	Type			
Saraphi, Chiang Mai	50	CR-39	Survey	21 (6)	Wanabongse et al.^39^
Chiang Mai	33 / 23	Ionization chamber	Case–Control	20 (15), 20.1 / 20.2 (p > 0.05)	Boonyaprapa et al.^40^
Chiang Mai	35/33	CR-39	Case–Control	57 (7)	Autsavapromporn et al.^30^
Doi Saket, Chiang Mai	30	CR-39	Survey	53 (15)	Thumvijit et al.^29^
Lampang	786	Activated charcoal	Survey	32 (21)	Tansurat et al.^41^
Thailand				16 (1.2)^g^	IAEA^28^
Global				39	WHO^19^
Upper northern Thailand	77 / 78	CR-39	Case–Control	105 (74), 109 / 102 (p = 0.033)	This study

g = geometric mean.

The indoor radon concentration can also vary as a result of other factors. Table 3 shows indoor radon concentrations and open window-to-wall ratios (ventilation) according to location of the bedroom, construction material of the ground and walls, and air conditioner use. This study found no significant differences between indoor radon concentration in the first and second floor of the bedroom location (p > 0.05). There was also no significant difference in indoor radon concentration regarding the walls or ground constructed with wood or concrete, which were the major materials of houses in UNT^42,43^.Many studies showed that different building materials contribute less than 20 Bq m^−3^ difference thus it does not enhance indoor radon concentrations^44^. In contrast, we found significant differences in open window-to-wall ratios’ ventilation in the houses.

**Table 3:** Arithmetic means indoor radon concentrations and open window-to-wall ratios depending on location, house construction materials, and air conditioner use.

House characteristics		Radon concentration		Open window-to-wall ratio	
		(Bq m^−3^)	p-value	(%)	p-value
Bedroom location	On 1st floor (79)	109 ± 82	0.42	25 ± 16	0.051
	On 2nd floor (66)	103 ± 78		29 ± 15	
Wall construction material	Cement (65)	112 ± 79	0.12	23 ± 15	0.004**
	Wood (80)	97 ± 75		30 ± 16	
Ground construction material	Cement (71)	113 ± 80	0.12	23 ± 14	0.005**
	Wood (71)	95 ± 73		31 ± 16	
Air conditioning	Yes (26)	146 ± 96	0.009**	14 ± 12	< 0.001**
	No (111)	99 ± 77		29 ± 15	

**Figure 2:**
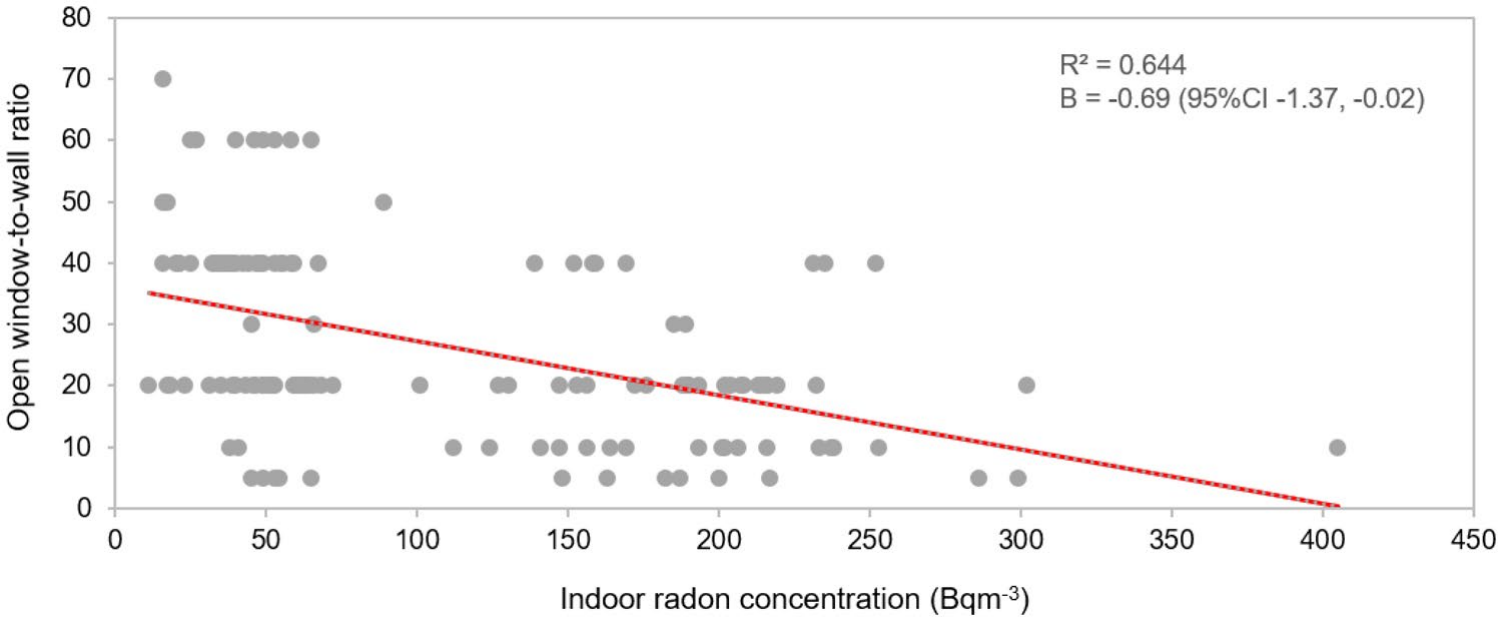
The association between open window-to-wall ratios and indoor radon concentrations in participant bedrooms.

The presence of an air conditioning in the room was associated with having significantly higher indoor radon concentrations. Generally, rooms with the air conditioning are likely better sealed to reduce outdoor air exchange and help control indoor air humidity and temperature. Consequently, this allows radon gas to accumulate and increase^45,46^. This means that the house characteristics might have not much influenced indoor radon concentration but air ventilation was more impactful. Figure 2 shows the association between open window-to-wall ratios and indoor radon concentrations. By adjusting with wall and ground materials, air conditioner use, geographical location of provinces and season of measurement. Every 10% increase in the open window-to-wall ratio was associated with a 6.9 Bq m^−3^ (B = −0.69, 95% CI −1.37, −0.02) decrease in indoor radon concentration. Thus, ventilation seems to be a factor with a greater influence on indoor radon concentrations than materials used in construction of the house.

Table 4 shows the average indoor radon concentration in the bedrooms of lung cancer cases compared to the healthy controls. By using the Wilcoxon paired test, the average concentration in case houses (109 ± 82 Bq m^−3^) was significantly higher (p = 0.033) than those of the control houses (103 ± 79 Bq m^−3^). As a result, radon may be a significant risk factor for development of lung cancer in UNT.

**Table 4:** Indoor radon concentrations (Bq m^−3^) and AED (annual effective dose) of lung cancer cases and healthy controls.

	All			Lung cancer case			Healthy controls			P-value
	n	Mean	SD	n	Mean	SD	n	Mean	SD	
Indoor radon (Bq m^−3^)	155	106	80	77	109	82	78	103	79	0.033*
AED (mSv y^−1^)	155	4.27	3.22	77	4.29	3.30	78	4.16	3.16	0.032*

In order to estimate the inhalation exposure of indoor radon, the annual effective dose (AED) was estimated. Participants spent between 12 to 24 h day^−1^ (average of 16.45 h day^−1^) indoors at home, which correlates to an indoor occupancy factor (*T*) of 0.69 (Table S1). This average, *T,* was used to estimate AED, which ranged between 0.44 and 12.18 mSv y^−1^ and an average of 4.27 mSv y^−1^, which is approximately 3 times higher than the global average AED of 1.3 mSv y^−1^^15^. This value is also higher than the previously reported measurements in Pa Miang, Chiang Mai province, where it ranged between 0.9—3.8 mSv y^−1^^29^. The AED of lung cancer cases was significantly higher than those of healthy controls (p = 0.032), with AED values of case and controls at 4.28 ± 3.0 and 4.11 ± 3.0 mSv y^−1^, respectively. This finding again suggests a role of radon exposure and the development of lung cancer in this region of Thailand.

The association between lung cancer and indoor radon, using indoor radon concentration less than 40 Bq m^−3^ as a reference level and adjusted by age, gender, smoking status, education and occupation was performed (Table S2). An association between radon exposure and lung cancer was restricted to males (OR = 4.60, 95% CI 1.00–21.09) and smokers (OR = 4.59, 95% CI 1.12–18.83) with indoor radon level 40–100 Bq m^−3^ only but not in overall groups (OR = 2.55, 95% CI 0.89–7.31 and OR = 1.79, 95% CI 0.66–4.87 for radon exposure at 40–100 Bq m^−3^ and more than 100 Bq m^−3^, respectively). Moreover, there was no association between higher exposure to indoor radon concentration. Therefore, the significant association found may be a result of chance^47^. Hence, EPA model^16^ and BEIR VI model^17^ were employed to estimate the number of lung cancer deaths attributable to indoor radon exposure in UNT. According to the Ministry of Public Health, Thailand, in 2015–2019, there were 10,164 lung cancer deaths in UNT, 6,115 males and 4,049 females. Table 5 shows the different exposures probably responsible for smoking, indoor radon exposure, the combination of smoking and indoor radon exposure for registered lung cancer deaths in UNT. Indoor radon exposure in UNT accounted for 26% and 28% of lung cancer deaths in males and females, respectively. Among these eight provinces, the highest lung cancer deaths attributable to indoor radon exposure was in Phrae province, at 37% of all lung cancer deaths, and the lowest was Lampang province (19%). The estimated number of lung cancer deaths due to radon exposure in male and female non-smokers was higher than those in smokers (Table 6). Since the sub-multiplicative interaction of smoking and radon were considered in the excess relative risk (ERR) calculation of the BEIR VI model that considered radon might be more influential in relative terms in non-smokers than in smokers^16,17^. These findings were consistent with other studies in several countries^48–51^. However, in our study approximately 96% of male lung cancer were smokers while 52% were female smokers (Table S3). Based on our study results, smoking is linked to a higher proportional risk of lung cancer death than radon exposure in males, but lower in females due to the higher male smokers than in females.

**Table 5:** The estimates of lung cancer deaths attributable to indoor radon exposure from 2015—2019 in the eight provinces of upper northern Thailand (UNT).

	Number of lung cancer deaths attributable to:										
	All	Only smoking		Smoking and radon		Only radon		Others		Radon	
	n	n	(%)	n	(%)	n	(%)	n	(%)	n	(%)
UNT	10,164	2580	25	571	6	2193	22	4820	47	2764	27
Male	6115	2093	34	475	8	1127	18	2420	40	1602	26
Female	4049	537	13	111	3	1017	25	2384	59	1128	28
Phrae	835	190	23	69	8	244	29	332	40	313	37
Male	548	168	31	62	11	136	25	182	33	198	36
Female	287	26	9	11	4	102	36	148	52	113	39
Phayao	998	343	34	116	12	219	22	320	32	335	34
Male	584	314	54	106	18	67	11	97	17	173	30
Female	414	30	7	16	4	142	34	226	55	158	38
Chiang Rai	1911	568	30	158	8	427	22	758	40	585	31
Male	1111	468	42	132	12	186	17	325	29	318	29
Female	800	132	17	36	5	225	28	407	51	261	33
Chiang Mai	2769	581	21	139	5	675	24	1374	50	814	29
Male	1572	417	27	102	6	353	22	700	45	455	29
Female	1197	165	14	38	3	319	27	675	56	357	30
Nan	869	153	18	29	3	190	22	497	57	219	25
Male	530	134	25	25	5	103	19	268	51	128	24
Female	339	40	12	4	1	86	25	209	62	90	27
Mae Hong Son	287	77	27	15	5	56	20	139	48	71	25
Male	183	50	27	10	5	36	20	87	48	46	25
Female	104	30	29	4	4	22	21	48	46	26	25
Lampang	1571	706	45	111	7	186	12	568	36	297	19
Male	1004	591	59	92	9	78	8	243	24	170	17
Female	567	117	21	19	3	108	19	323	57	127	22
Lamphun	924	142	15	24	3	195	21	563	61	219	24
Male	583	130	22	22	4	112	19	319	55	134	23
Female	341	27	8	4	1	79	23	231	68	83	24

**Table 6:** The estimates of lung cancer deaths attributable to indoor radon exposure in 2015—2019 for eight provinces of upper northern Thailand (UNT) by gender and smoking status, according to the EAC models.

Provinces	Number of lung cancer deaths attributable to indoor radon exposure											
	All				Male				Female			
	Smoker		Non-smoker		Smoker		Non-smoker		Smoker		Non-smoker	
	n	%	n	%	n	%	n	%	n	%	n	%
UNT	405	18	2479	31	418	18	1224	32	63	17	1101	30
Phrae	42	27	286	42	49	27	157	43	5	14	111	42
Phayao	65	25	300	41	60	25	140	41	11	25	149	40
Chiang Rai	95	22	530	36	93	22	250	36	15	9	259	36
Chiang Mai	134	19	684	33	124	20	316	34	25	19	342	32
Nan	26	16	195	28	26	16	102	28	4	8	86	27
Mae Hong Son	11	16	63	29	12	17	33	29	2	5	26	28
Lampang	214	14	306	25	46	13	162	24	7	14	130	25
Lamphun	30	14	183	26	32	14	94	26	4	14	79	26

Previous studies have estimated that about 4–29% of all lung cancer deaths were attributable to radon exposure, depending on radon concentration and the employed model^52,53^. Table 7 shows the comparison percentage of lung cancer deaths attributable to radon with previous studies calculated using the exposure-age concentration (EAC) model. The percentage varied depending on indoor radon concentrations and smoking status in males and females in each population.

**Table 7:** Comparison percentage of lung cancer deaths attributable to indoor radon in previous studies using the exposure-age-concentration model (EAC) of BEIR VI.

Country	Average indoor radon (Bqm^−3^)	Lung cancer deaths attributable to indoor radon (%)			References
		Male	Female	Total	
USA	46	14.1	15.3	13.9	BEIR VI^16^
Canada	43			13.6	Peterson et al.^46^
France	89			13	Catelinois et al.^44^
Portugal	81	27	34		Veloso et al.^52^
South Korea	62	19.5	28.2		Lee et al.^47^
Thailand	16			9.4	Gaskin et al.^49^
UNT Thailand	105	26	28	27	This study

According to our estimation, approximately 553 lung cancer deaths every year were attributable to indoor radon exposure in UNT between 2015 and 2019. This result is high relative to the total lung cancer deaths in Thailand estimated to be attributable to radon, which was 1,660 cases in 2012^53^. Thus, approximately one third of lung cancer deaths attributable to indoor radon exposure in Thailand was in UNT. The higher attributable risk in this study is due to the higher indoor radon concentration measured in UNT than the national average. However, these values were in a worldwide range between 3 and 40% of all lung cancer deaths due to indoor radon exposure^52,53^. These values tend to increase in high radon countries.

Our study found that there was significantly higher smoking behavior among lung cancer cases (80%) than healthy controls (52%) (p > 0.001) (Table S3). Smoking is the primary causal development of lung cancer worldwide^11^^.^ Reportedly, smoking has a synergistic effect with high radon concentration to increase lung cancer risk by up to 25 times^17,23^. However, in northern Thailand, smoking prevalence was the lowest (11.3%) than those of the other part and the country mean (15.2%)^9^ while lung cancer incidence was the highest prevalence^3,5^. Further, the attributable risk of only smoking and smoking with radon of lung cancer in this region of Thailand also needs to be elucidated. Greater than 40% of houses in this study had radon levels higher than the recommended activity level of WHO as 100 Bq m^−3^.

The higher indoor radon value in lung cancer cases compared to those of healthy controls in this study suggests that radon may be a risk factor for development of lung cancer in UNT. This may be synergistic in effect with other factors such as smoking to increase the high incidence and mortality of lung cancer in this area. In UNT, open biomass burning, primarily for agricultural purposes, also results in high ambient air pollution, which may further contribute to the increased risk of lung cancer in this region^14,54–56^.

## Conclusion

Lung cancer is one of the major health burdens in UNT. High levels of residential radon can increase the risk of lung cancer in the general population, and these levels are influenced by different geological and topographic characteristics, along with house ventilation. In eight provinces of UNT, the measured indoor radon concentration ranged from 11–405 Bq m^−3^, corresponding to an annual effective dose of 0.44–12.18 mSv y^−1^. The mean, which exceeded the global mean, and greater than 41% of houses in this study had higher indoor radon concentrations than the WHO recommended level (100 Bq m^−3^). The finding of higher indoor radon concentrations in the houses of lung cancer patients compared to those of healthy controls suggests a contribution of indoor radon to lung cancer in this region. The EAC model of BEIR VI estimated that 27% of all lung cancer deaths were attributable to residential radon exposure or approximately 553 lung cancer deaths per year. Indoor radon may be responsible for a substantial proportion of lung cancer deaths in UNT, and an effective strategy to prevent and mitigate indoor radon exposure is needed to reduce the high lung cancer mortality in UNT.

## Methods

### Study design

This study was conducted in eight provinces of UNT. The study process included field measurement and data collection from participants. The primary lung cancer patients were enrolled from hospitals while the healthy controls were enrolled at the same communities of lung cancer cases who had no history of lung cancer in family members. All participants must have lived in UNT at least 5 years. All participants were informed about the study information, including risk or any inconveniences that may have occurred from the study. Informed consent was obtained from all participants prior to enrollment. All experiment protocols and ethical clearance were approved from the Human Experimentation Committee, Research Institute for Health Sciences (Study code: Project No. 1/59, approved on 10 May 2016) and the Research Ethics Committee, Faculty of Medicine, Chiang Mai University (Study code: NONE-2558-03633, approved on 20 July 2016).

### Study area

This study area is located in UNT that covers approximately 82,500 km^2^ and comprises 8 provinces including Chiang Mai, Chiang Rai, Lamphun, Lampang, Phayao, Nan, Phrae and Mae Hong Son province (Fig. 1a). UNT consists of basins of the 4 main rivers namely Ping, Wang, Yom, and Nan and 9 active fault zones. There are also basins surrounded by the mountains^57,58^.

### Data collection

From September 2018 to December 2020, seventy-seven lung cancer cases and 78 healthy controls matched by sex and age (± 5 years) who lived within a 5 km radius of lung cancer cases were enrolled into this study with the inclusion criteria as Supplementary Table S4. All participants were interviewed by questionnaire about individual data, history of smoking, occupation, lifestyle, and house characteristics (construction materials, bedroom location and ventilation). Ventilation in the bedroom was estimated using an open window-to-wall ratio that refers to the percentage of the open area of the window or vent in the wall to the gross wall area of the room (Fig. S2).

### Indoor radon measurements

Participants' houses were located on a map of UNT which was 50 × 50 km gridded. The empty grids where no participant houses installed the radon detectors, additional, 37 new houses were enrolled to the extra heathy control. 192 houses (houses of the 77 lung cancer cases and those of the 115 healthy controls) underwent indoor radon measurement (Fig. S1). The indoor radon concentration was determined using a closed alpha-track detector that contained electrically conducting plastic film of allyl diglycol carbonate (CR-39/PADC) using the Radtrak system manufactured by Radonova Laboratory AB, (Uppsala, Sweden). From February 2019 to February 2021, a total of 192 CR-39 detectors were placed in the bedrooms of all participants, installing the units away from windows, doors, electric devices or heat sources, and at least 20 cm away from the wall and 1 m above the floor. The detectors were installed for 3 months to measure indoor radon concentration. Then, the detectors were individually packed in ziplock plastic bags and placed in the large bag, shipped and measured by the Radonova Laboratory AB. On the film, the alpha particles make small tracks which are enlarged with chemical etching and later counted in a microscope using a state-of-the-art image scanner to determine the radon concentration by ISO 17,025 accredited system with an uncertainty of 6% at 200 Bq m^−3^ (source: https://radonovalaboratories.com).

### Statistical analysis and health risk assessments

Radon gas can be inhaled through the respiratory tract and interact with biological molecules in the lung leading to lung cancer by damaging DNA, which is a potential health risk. Thus, to evaluate the exposure doses received for indoor radon inhalation, the annual effective dose (AED) in the unit of.

mSv y^−1^ was estimated using following the equation:

1 $$AED = C_{Rn} \times F \, \times \, T \, \times \, D$$

where *C*_*Rn*_ is the radon concentration (Bq m^−3^), *F* is the equilibrium factor of radon and its daughters which is equal to 0.4 for indoors^17^, *T* is the occupancy time and *D* is the dose conversion coefficient, *FD* can merge into the dose conversion factor equal to 6.7 × 10^–6^ mSv/Bq h m^−3^ for indoor radon^59^.

The number of lung cancer deaths attributable to radon exposure in this region was estimated using Eq. (2)

2 $$N_{r,a} = \, \left( {ERR \times N_{a} } \right) \, / \, \left( {{ 1 } + ERR} \right)$$

where N_*r,a*_ is the lung cancer deaths attributable to *r* radon exposure at attained age *a,* ERR is the excess relative risk at attained age *a* and exposure *r,* and *N* is the number of lung cancer deaths at attained age *a.*

The excess relative risk (ERR) can be calculated following the exposure-age concentration (EAC) model from BEIR VI^17^ and some parameters were received from EPA model^16^ that giving as follows the Eq. (3) by assumed that all individuals in the same provinces were equally exposed and the concentrations that exposed were unchanged over their lifetime.

3 $$ERR = \, \beta \, \left( {w{5}_{{ - {14}}} + \, 0.{78}w_{{{15} - {24}}} + \, 0.{51}w_{{{25} + }} } \right)$$

where β is the exposure–response parameter or risk coefficient that equal to 6.9 × 10^–3^ for attained age greater than 75 years old, *w* is the exposure windows, *w*_*5-14*_*, w*_*15-24*_ and *w*_*25*+_ define the exposure rate incurred between *5-14y, 15–24 y* and more than *25 y* before the current age, respectively.

## Supplementary Information

Supplementary Information.
