# Supplementary material for: Estimation of lung cancer deaths attributable to indoor radon exposure in upper northern Thailand
Source: Sci Rep. 2022 Mar 25;12:5169. doi: 10.1038/s41598-022-09122-y (PMC8956686; doi:10.1038/s41598-022-09122-y)
Supplement: Supplementary file 1 — Supplementary Information. [file 41598_2022_9122_MOESM1_ESM.docx]

**SUPPLEMENTARY IMFORMATION**

**Estimation of lung cancer deaths attributable to indoor radon exposure in**

**upper northern Thailand**

Kawinwut Somsunun^1, 2^, Tippawan Prapamontol^1^*, Chaicharn Pothirat^3^, Chalerm Liwsrisakun^3^, Donsuk Pongnikorn^4^, Duriya Fongmoon^4^, Somporn Chantara^5^, Rawiwan Wongpoomchai^6^, Warangkana Naksen^7^, Narongchai Autsavapromporn^8^ & Shinji Tokonami^9^*

^1^ Environment and Health Research Unit, Research Institute for Health Sciences (RIHES), Chiang Mai University, Chiang Mai 50200, Thailand;

^2^ PhD Degree Program in Environmental Science, Environmental Science Research Center, Faculty of Science, Chiang Mai University, Chiang Mai 50200, Thailand;

^3^ Department of Internal Medicine, Faculty of Medicine, Chiang Mai University, Chiang Mai 50200, Thailand;

^4^ Lampang Cancer Hospital, Department of Medical Services, Ministry of Public Health, Lampang, Thailand;

^5^ Environmental Science Research Center, Faculty of Science, Chiang Mai University, Chiang Mai 50200, Thailand;

^6^ Department of Biochemistry, Faculty of Medicine, Chiang Mai University, Chiang Mai 50200, Thailand;

^7^ Faculty of Public Health, Chiang Mai University, Chiang Mai 50200, Thailand;

^8^ Department of Radiology, Faculty of Medicine, Chiang Mai University, Chiang Mai 50200, Thailand;

^9^ Institute of Radiation Emergency Medicine, Hirosaki University, Hirosaki, Aomori 036-8564, Japan

*Corresponding author

Tippawan Prapamontol

E-mail: tippawan.prapamontol@cmu.ac.th

**Supplementary table S1.** Demographic characteristics of lung cancer cases and healthy controls.

|  | | Lung cancer cases (77) | Healthy controls (78) |
| --- | --- | --- | --- |
| Age, mean (range) | | | |
|  |  | 60 (22–78) | 60 (25–78) |
| Occupancy time (Spend time in home, h day^-1^), mean (range) | | | |
|  |  | 16.1 (12–23) | 16.8 (14–24) |
| Sex, n (%) | | | |
|  | Male | 50 (65) | 48 (62) |
|  | Female | 27 (35) | 29 (38) |
| Smoking, n (%) | | | |
|  | Current smoker | 32 (42) | 17 (22) |
|  | Former smoker | 29 (38) | 23 (30) |
|  | Non-smoker | 15 (20) | 37 (48) |
| Occupation, n (%) | | | |
|  | Farm worker | 46 (61) | 57 (74) |
|  | Non- farm worker | 29 (39) | 20 (26) |
| Residential provinces, n (%) | | | |
|  | Chiang Mai | 19 (25) | 17 (22) |
|  | Lamphun | 19 (25) | 20 (26) |
|  | Lampang | 16 (21) | 16 (20) |
|  | Chiang Rai | 10 (13) | 11 (14) |
|  | Phayao | 3 (4) | 2 (3) |
|  | Phrae | 5 (6) | 7 (9) |
|  | Nan | 1 (1) | 0 (0) |
|  | Mae Hong Son | 4 (5) | 5 (6) |
| Residential radon (Bq m^-3)^, n (%) | | | |
|  | <100 (WHO recommended level) | 44 (57) | 46 (59) |
|  | >=100 | 33 (43) | 32 (41) |
|  | <148 (EPA recommended level) | 53 (69) | 52 (67) |
|  | >=148 | 24 (31) | 26 (33) |

**Supplementary table S2.** Odds ratio (ORs) and 95% confidence intervals by indoor radon concentration (Bq m^-3^) adjusted for age, gender, smoking, education and occupation and regarding with gender and smoking status.

| Indoor radon  (Bq m^-3^) | n (Case/  Control) | OR (95% CI) | n (Case/  Control) | OR (95% CI) | n (Case/  Control) | OR (95% CI) |
| --- | --- | --- | --- | --- | --- | --- |
| Gender | | | | | | |
|  | All | | Male | | Female | |
| < 40 | 12/22 | 1 | 8/15 | 1 | 4/7 | 1 |
| 40-100 | 32/24 | 2.55 (0.89-7.31) | 20/13 | 4.60 (1.00-21.09)* | 12/11 | 0.76 (0.11-5.25) |
| >100 | 24/25 | 1.79 (0.66-4.87) | 17/15 | 2.23 (0.61-8.09) | 7/10 | 1.15 (0.17-8.02) |
| Smoking status | | | | | | |
|  | All | | Non-smoker | | Smoker | |
| < 40 | 12/22 | 1 | 4/10 | 1 | 7/12 | 1 |
| 40-100 | 32/24 | 2.55 (0.89-7.31) | 5/12 | 0.90 (0.13-6.17) | 27/12 | 4.59 (1.12-18.83)* |
| >100 | 24/25 | 1.79 (0.66-4.87) | 4/11 | 1.28 (0.19-8.69) | 20/14 | 2.51 (0.70-9.15) |

**Supplementary table S3.** Number of smoker and non-smoker in lung cancer cases and healthy controls.

| Smoking status | Lung cancer cases, n (%) | | Healthy controls, n (%) | |
| --- | --- | --- | --- | --- |
|  | Male | Female | Male | Female |
| Non-smoker | 2(4.1) | 13(48.1) | 13(27.1) | 24(82.8) |
| Smoker | 47(95.6) | 14(51.9) | 35(72.9) | 5(17.2) |
| Non-smoker | 15(19.7) | | 37(48.1) | |
| Smoker | 61(80.3) | | 40(51.9) | |

**Supplementary figure S1.** The enrolment protocol and criteria of participants.


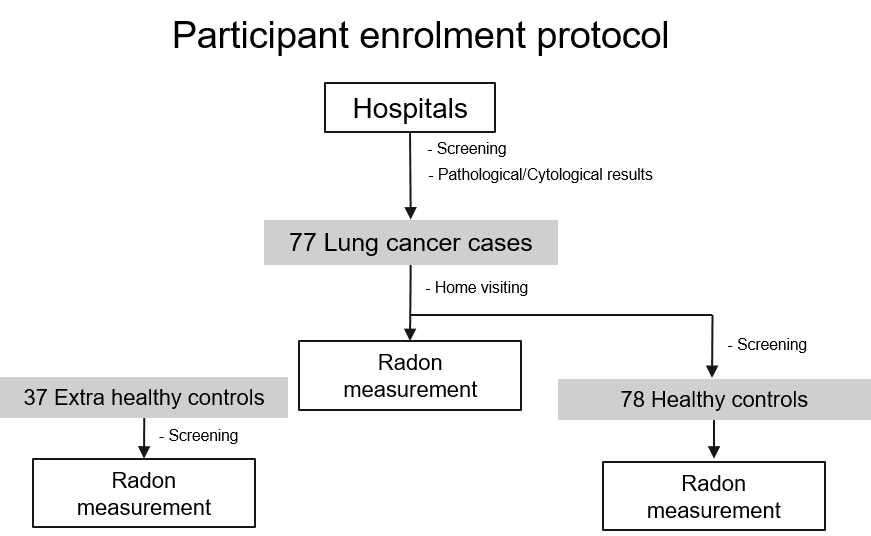


**Supplementary table S4.** Criteria of participant recruitment.

| Criteria of participants | Lung cancer cases | Healthy controls | Extra  healthy controls |
| --- | --- | --- | --- |
| 1. Age between 18 and 80 years old. | √ | √ | √ |
| 2. Primary lung cancer. (pathologically/cytologically confirmed) | √ |  |  |
| 3. No non-communicable diseases (NCDs) such as diabetes, hypertension and some infectious diseases such as tuberculosis. | √ | √ |  |
| 4. Lived in eight provinces of upper northern Thailand at least 5 years continuously. | √ | √ | √ |
| 5. Matched by sex and age (± 5 years) with lung cancer cases in the same community (5 km radius) |  | √ |  |
| 6. No lung cancer in family members |  | √ | √ |
| 7. No any other cancers. | √ | √ | √ |

**Supplementary figure S2.** The estimation of ventilation in terms of open window-to-wall ratio; the white area represents the open area of window or vent in the wall of the room, the square blue area represents the whole wall area of the room.


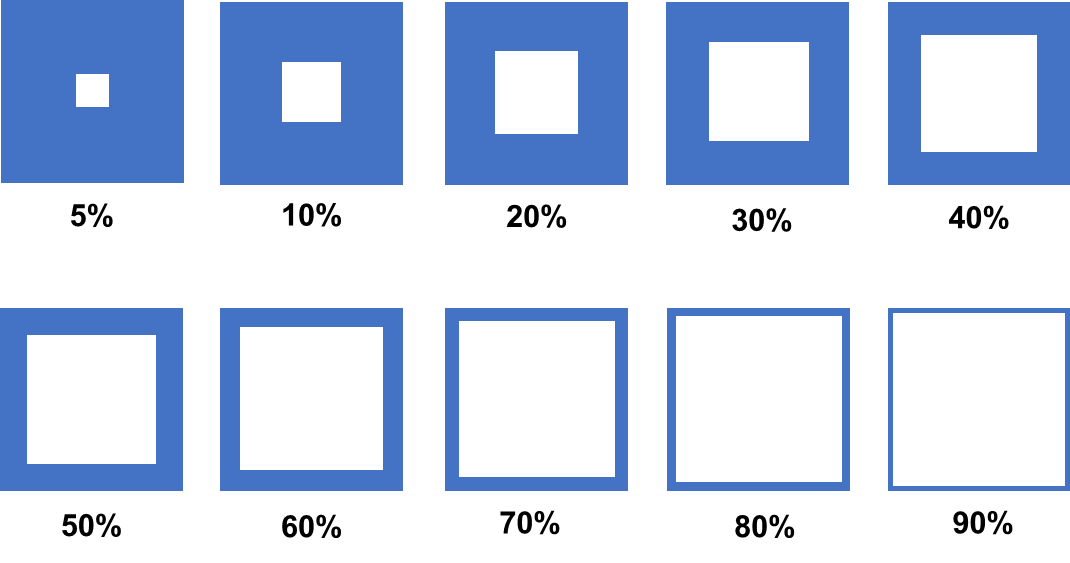


Open window-to-wall ratio was applied from the calculation of window-to-wall ratio. It was referred to the percentage of only the open area of the window or vent in the wall to the gross wall area of the room by following the equation:

$$Open window to wall ratio= \frac{Net open area in the wall}{Gross wall area} x 100$$
